# Flight Path Setting and Data Quality Assessments for Unmanned-Aerial-Vehicle-Based Photogrammetric Bridge Deck Documentation

**DOI:** 10.3390/s23167159

**Published:** 2023-08-14

**Authors:** Siyuan Chen, Xiangding Zeng, Debra F. Laefer, Linh Truong-Hong, Eleni Mangina

**Affiliations:** 1 School of Information Science and Engineering, Hunan Institute of Science and Technology, Yueyang 414015, China; 2 School of Civil Engineering, University College Dublin, D04C1P1 Dublin, Ireland; debra.laefer@nyu.edu; 3 College of Mechanical Engineering, Hunan Institute of Science and Technology, Yueyang 414015, China; xiangdingzeng@vip.hnist.edu.cn; 4 Center for Urban Science and Progress, Tandon School for Engineering, New York University, New York, NY 10012, USA; 5 School of Civil Engineering, Technical University Delft, 2628 CD Delft, The Netherlands; l.truong@tudelft.nl; 6 School of Computer Science, University College Dublin, D04C1P1 Dublin, Ireland; eleni.mangina@ucd.ie

**Keywords:** UAV, SFM, photogrammetry, point cloud, quality evaluation

## Abstract

Imagery from Unmanned Aerial Vehicles can be used to generate three-dimensional (3D) point cloud models. However, final data quality is impacted by the flight altitude, camera angle, overlap rate, and data processing strategies. Typically, both overview images and redundant close-range images are collected, which significantly increases the data collection and processing time. To investigate the relationship between input resources and output quality, a suite of seven metrics is proposed including total points, average point density, uniformity, yield rate, coverage, geometry accuracy, and time efficiency. When applied in the field to a full-scale structure, the UAV altitude and camera angle most strongly affected data density and uniformity. A 66% overlapping was needed for successful 3D reconstruction. Conducting multiple flight paths improved local geometric accuracy better than increasing the overlapping rate. The highest coverage was achieved at 77% due to the formation of semi-irregular gridded gaps between point groups as an artefact of the Structure from Motion process. No single set of flight parameters was optimal for every data collection goal. Hence, understanding flight path parameter impacts is crucial to optimal UAV data collection.

## 1. Introduction

To ensure ongoing serviceability and safety, bridges must be inspected periodically as per local regulations (e.g., AASHTO, 1970 [1]; RAIU, 2010 [2]). Although many methods have been developed to support bridge inspection, visual inspection using on-site inspectors dominates. However, the visual inspection has many shortcomings, including the following aspects: (1) subjective results; (2) access only via heavy and/or specialty equipment; (3) traffic closures; (4) the requirement for highly skilled trained inspectors; (5) safety risks for inspectors; and (6) time-consuming and expensive processes. These aspects are particularly challenging in the absence of as-built drawings or an existing 3D model.

Bridge documentation and inspection been conducted using cameras (Xie et al., 2018) [3] and/or laser scanners (Truong-Hong and Laefer, 2015 [4]; Gyetvai et al., 2018 [5]), and even microwave radar interferometry (Zhang et al., 2018) [6] and synthetic aperture radar. Three-dimensional point clouds can be produced either directly through laser scanning or indirectly through assembling two-dimensional images. However, the quality of these point clouds is highly related to the view angles and offset distances. For example, the camera or scanner is set on the bridge deck or river bank, and incomplete coverage of the entire structure may occur due to the fixed field of view and positioning logistics. Low-cost UAVs equipped with cameras provide workarounds and offer many benefits such as non-contact measurement, avoidance of traffic closures, and use of non-specialized equipment (Atole et al., 2017) [7], while providing better data coverage in hard-to-reach areas like beneath the deck or the upper portions of a bridge’s pylons (Chen et al., 2019) [8].

As an alternative to laser scanners, low-cost UAVs equipped with a single digital camera can generate dense and accurate point clouds when coupled with state-of-the-art computer-vision-based methods. Such capabilities have accelerated the adoption of UAV-based data capture for a wide range of infrastructure needs including building modelling (Byrne and Laefer, 2016) [9], dam inspection (Sizeng Zhao et al., 2021) [10], construction site monitoring (Hoegner et al., 2016) [11], and road surface evaluation (Chen and Dou, 2018) [12]. However, there are many factors that directly influence the model’s final accuracy, resolution, and completeness. While these are known to include the camera positioning, number of images collected, overlap extent, and image quality, the interaction between these factors with respect to their impact on the final 3D model quality has yet to be quantified. Additionally, to explore their capability for comprehensive documentation and to devise optimization strategies, a series of reliable and systematic evaluation metrics are required to evaluate the results. To date, these have yet to be established. Therefore, this paper introduces four data quality evaluation metrics for bridge deck or roadway point clouds and investigates the interaction between flight path parameters and the quality of the reconstructed point cloud using those metrics and with respect to a terrestrial laser scanner.

## 2. Background

In recent years, with the improvement in design, control, and navigation technologies, UAVs are becoming cheaper and more easily accessible (Chen et al., 2016) [13]. In addition to conventional fixed-wing UAVs, newer designs developed for low-altitude, close-range inspection are increasingly available. For example, multirotor UAVs with outstanding hovering capabilities and better safety tolerances for rotor failure are already being used to a limited extent for civil infrastructure inspection (Liu et al., 2014) [14]. The incorporation of navigation sensors, such as Inertial Measurement Units (IMUs) (Li et al., 2015) [15], and obstacle detection sensors, such as optical flow cameras (Honegger et al., 2013) [16] and ultrasonic sensors (Papa and Del, 2015) [17], is further improving UAV reliability. For data collection purposes, laser scanners (Chisholm et al., 2013) [18] and digital cameras (Ferrick et al., 2012) [19] are commonly used separately and together both with and without UAVs. Examples are shown in Table 1.

Laser scanning provides high-quality 3D point clouds but the equipment is comparatively expensive. Imagery is arguably more cost effective but not without difficulties, especially as many applications including full documentation and crack detection require 3D data (Chen et al., 2011) [36]. To obtain depth information from 2D images, they must be stitched together to form either stereoscopic images or a point cloud. For UAV inspection, the latter is commonly achieved using the SfM (Structure from Motion) method. The approach relies on having overlapping images taken from multiple viewpoints to enable the formation of a 3D point cloud. The process starts by detecting key points in each image through which images can be linked. This procedure can be accomplished by applying feature detectors like scale invariant feature transforms (SIFT) (Lowe, 1999) [37] or the speeded up robust features (SURF) method (Bay et al., 2008) [38]. Then, the 3D structure and camera motion based on the extracted features can be estimated to improve triangulation. Subsequently, a spare bundle adjustment (Lourakis and Argyros, 2009) [39] can be used to optimize the camera’s position and generate a sparse point cloud to represent the object. Finally, point density can be intensified by applying multi-view stereo (MSV) techniques (Yasutaka and Hernández, 2015) [40]. Many of these procedures and related algorithms appear singly or in combination with many off-the-shelf software products, including Agisoft Photoscan, Pix4D, OpenMVG, and VisualSfM. For the sake of simplicity, in this paper the term SfM will be used to denote the entire reconstruction procedure.

While SfM is well established, the presence of cars, shadows, and specific terrains can complicate the subsequent data processing. The resulting 3D point clouds are also impacted by camera setting, lens distortion, flying height, quality and quantity of images, distribution of perspectives in those images, and capture angles (Smith and Vericat, 2015) [41]. Recent efforts have investigated the impact of these factors on the quality and quantity of SfM-generated point clouds. For example, Byrne et al. (2017) [42] studied the effects of camera mode and lens settings on point density. This study showed that the lens distortion under a wide view mode generated a point cloud only half as dense as the one derived from images with no distortion. Similarly, poor data density and distortions were observed by Chen et al. (2017) [43] under laboratory conditions when the angle of incidence was high. That study recommended combining images from different oblique angles (e.g., 45° with 60°) to minimize the density and distortion issues that appear when they are processed separately. A similar recommendation was made previously by James et al. (2014) [44], where the addition of oblique or parallel images was performed to reduce the error in the digital elevation models by as much as two orders of magnitude. However, all images may not be equally valuable. For example, Dandois et al. (2015) [45] found that denser point clouds were more easily produced on cloudy days due to the absence the unwanted shadows produced on sunny days. However, Chen et al. (2017) demonstrated that under laboratory conditions direct light increased the contrast in the images, which improved model accuracy, thereby implying that sunny days will lead to more accurate point clouds even though they may be less dense than those collected on cloudy days. Han et al. (2023) [35] conducted a study on the influence of UAV flight paths on the geometric accuracy of the final model. However, it is important to note that the geometric accuracy of the point cloud does not solely represent the point cloud quality. In real-world engineering scenarios, the point cloud quality typically requires evaluation from various perspectives, including volume density, completeness, geometric accuracy, and time taken, among others.

Although the aforementioned studies have recognized the effect of some variables related to camera calibration or data post-processing, a systematic understanding of how flight path parameters affect final point cloud quality has yet to be established, especially at lower altitudes (below 50 m) and in the presence of buildings and other infrastructure. Furthermore, there has yet to emerge a standard evaluation process for SfM point clouds. While some studies, such as those by Byrne et al. (2017) [46] and Slocum and Parrish (2017) [47], used the final number of points in a point cloud or point density as a proxy for quality, this is not widely performed, and while a few researchers (e.g., (Dandois et al., 2015)) have considered the geometric accuracy of the reconstructed point cloud with respect to GPS and GCP, these properties do not address data completeness or uniformity. To address these knowledge gaps, a systematic evaluation method is proposed in this paper to quantitatively study image-based point clouds. The usefulness of these metrics is then demonstrated as a means to determine the impact of flight parameters on 3D point cloud reconstructions.

## 3. Quality-Based Evaluation Metrics

To determine the quality of reconstructed point clouds for bridge deck and road surface documentation, a quintet of new quality-based point cloud evaluation metrics is herein proposed that covers the following aspects: (i) point average density, (ii) uniformity, (iii) completeness, (iv) overall point yield, and (v) geometric accuracy. Having these objective metrics will then enable more informed decisions about UAV flight path planning with respect to the required outputs. Each of these metrics is described in this section and then implemented in the next section as part of an actual field study.

### 3.1. Point Density and Uniformity

The first two proposed evaluation metrics are overall point density and point uniformity. Point cloud density is an indicator of data resolution. When the overall density is too low, small details will not appear in the dataset and may preclude damage identification because of poor data availability. Conversely, overly dense point clouds will have redundant data, thereby unnecessarily requiring storage space and slowing analyses. Non-uniform point clouds will include both high-density and low-density areas.

These defects influence the quality of subsequent data processing and the affiliated outputs as well. This may include the performance of neighbor search algorithms and feature estimation processes, further data simplification (Moenning and Dodgson, 2003) [48], surface reconstruction (Huang et al., 2009 [49]; Holz and Behnke, 2014 [50]), and multi-dataset registration (Holz and Behnke, 2014; Huang et al., 2009). In addition, employing algorithms that specify a minimum-density threshold (Zolanvari and Laefer, 2016 [51]; Truong-Hong et al., 2013 [52]) may be especially challenging as even quantification of the minimum density would require significant resources to establish. Unfortunately, in real surveys, both TLS (terrestrial laser scanning) point clouds and imagery-derived point clouds (referred to here as SfM point clouds) are non-uniform.

To overcome these aforementioned problems, identifying the parameters that most affect the density and uniformity of a point cloud is necessary. Due to different data capturing mechanisms, parameters impacting the TLS and SfM point clouds differ. The non-uniformity of TLS data is directly linked to offset distance and the angle of incidence, as well as data capture speed. Specifically, smaller offsets and incidence angles tend to produce higher densities and lower differences in data distributions and can be represented as largely linear relationships (Laefer et al., 2009 [53]; Quagliarini et al., 2017 [54]). However, the main factors contributing to non-uniformity in SfM point clouds are less understood, and the explicit relationship between image resolution and overlapping rate has yet to be studied systematically.

**Figure 1:**
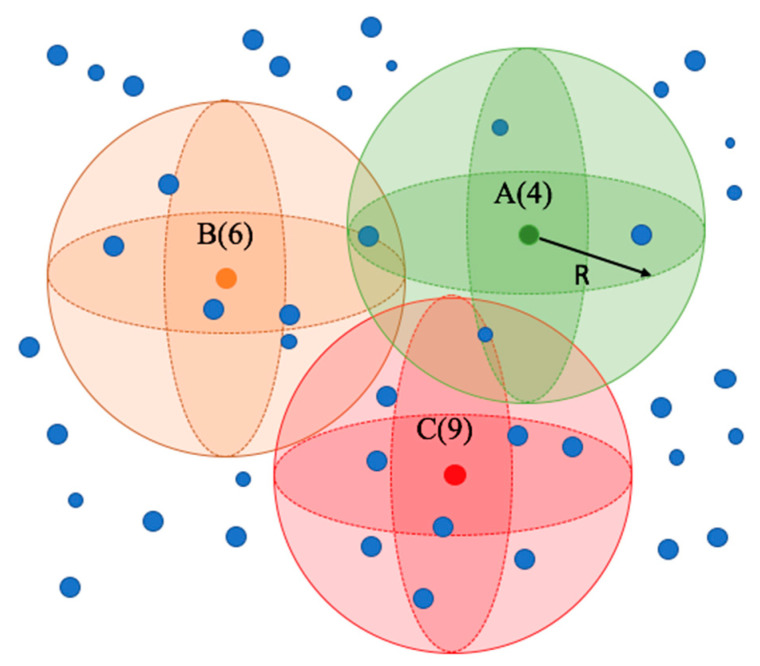
Volume density.

To identify the critical data capture parameters that affect data densities and uniformities in SfM point clouds collected from UAVs, a volume density calculation is proposed; volume density is more representative as the surfaces are not entirely flat. The approach considers point distribution across a sphere. As shown in Figure 1, for each point P_i_, the number of neighbour points inside a specified spherical neighbourhood (N_i_) with a radius R is calculated using a k-Nearest Neighbour (kNN) algorithm (Fukunaga and Hostetler, 1973) [55]. The volume density of Pi is equal to *N_i_* divided by the neighbourhood volume. As such, the general density can be represented by the statistical characteristics of each point. As shown in Equation (1), the average density (AD) will represent the overall density of the point cloud, while a standard deviation (SD) (Equation (2)) is used to evaluate its uniformity level. As density may vary greatly between datasets, a direct comparison of the SD is not meaningful. Thus, the term relative standard deviation (RSD) is introduced in the form of Equation (3) as an indication of the uniformity. Lower RSD values represent more uniform datasets.

(1) $$D=\frac{1}{n}\sum_{i=1}^{n}\frac{N_{i}}{4/3\pi R^{3}}$$

(2) $$SD=\sqrt{\frac{1}{n}\sum_{i=1}^{n}{\left(\frac{N_{i}}{4/3\pi R^{3}}-AD\right)}^{2}}$$

(3) $$RSD=\frac{SD}{AD}\times 100\%$$

### 3.2. Completeness

**Figure 2:**
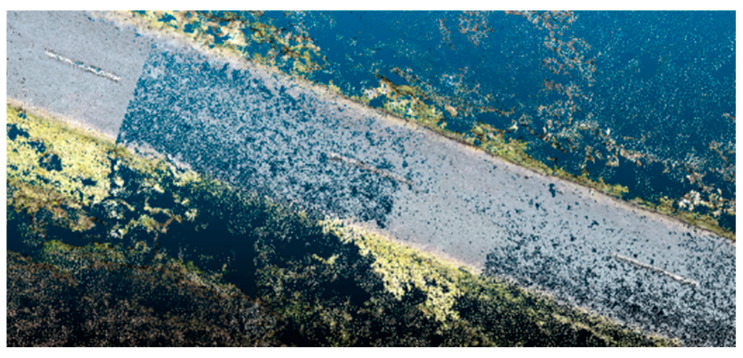
Incomplete dataset in SfM roadway point cloud.

The third metric relates to completeness. Incompleteness in SfM point clouds commonly relates to insufficient coverage, insufficient overlap, inability to discern textures in the images, and overall poor image quality. As shown in Figure 2, the missing data appear as either missing patches or randomly distributed empty spots. In contrast, incompleteness in TLS datasets is usually caused by high angles of incidence or line-of-sight interference, both common artefacts of site access issues.

**Figure 3:**
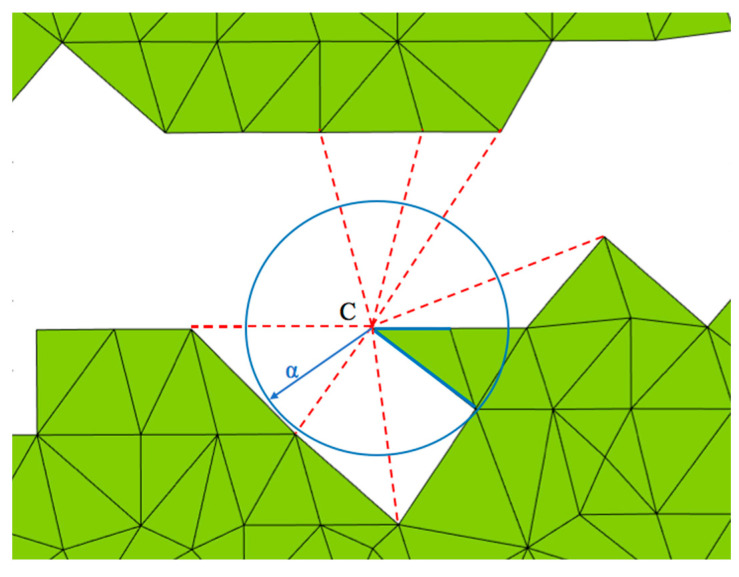
Mesh generation.

To quantify the completeness of a point cloud, a mesh-based area calculation method is introduced. Since the bridge deck upper surface is nearly a flat plane, to make the calculation more efficient, a 2D mesh is used. The process involves first projecting the data points onto a normal plane. Then, a triangulation mesh is built from the projected data points based on x and y coordinates across an entire plane. Next, the threshold radius α is applied to control the searching radius for the mesh generation. For any point C within the radius α, if a neighbour point exists, a triangular mesh will be generated, as shown in Figure 3. The mesh is then used to calculate the area. Thus, by controlling the threshold α, the areas with and without coverage can be calculated.

**Figure 4:**
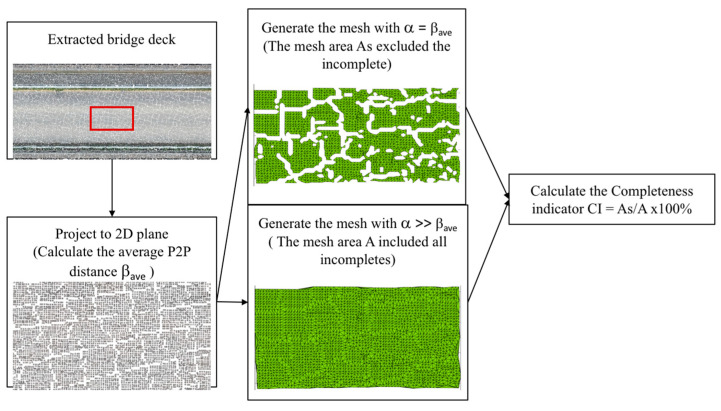
Workflow for completeness evaluation.

To choose an appropriate α, the average distance of any point to its nearest neighbours must be measured. In this algorithm, points are randomly taken from the original data as querying points and used in a KNN search to find the closest point to each query point. Then, the average Euclidean distance (β_ave_) of all pairs of query points and their closest neighbour are calculated. If the α value is close to or equal to the β_ave_, then the mesh will overlook the incomplete areas and only represent the real data coverage. Instead, if α is set as much larger than the β_ave_, the mesh will connect all points and measure the entirety of the pavement. By comparing these two meshes, the degree of coverage for each dataset can be measured and compared, as shown in Figure 4. The completeness index (CI) is equal to the percentage of the area covered by the points compared to the entirety of the area enveloped inside the boundary, as shown in Equation (4).

(4) $$CI=\frac{A_{s}}{A}\times 100\%$$

In an ideal world, the smallest known feature or damage could be used, but that would require a priori knowledge or extensive pre-processing and localized surface generation prior to implementation of this check.

### 3.3. Geometric Accuracy

The fourth evaluation metric is geometric accuracy, which is important for engineering inspection, especially for applications such as deformation monitoring and quantifiable damage assessment. In a surveying context, Lucieer et al. (2014) [56] compared a UAV-based SfM point cloud to checks with GPS readings through a set of 24 GCPs for landslide mapping. Similarly, Mosbrucker et al. (2017) [57] used LiDAR-derived digital terrain models as the ground truth along with 103 control points for topographic mapping. Such methods rely on GCPs for the large-scale global accuracy assessment and demonstrated a range of SfM-point cloud accuracy from 0.05 m to 0.97 m for the applications and equipment considered in those studies. This type of approach works well for topographic surveying, as the goal is to compare the positioning of data points to known positions in the real world.

For documentation, inspection, and modelling, however, the accuracy must be tied to the geometric object under evaluation. For small-scale surveys, Palmer et al. (2015) [58] used TLS data as the ground truth. In that process, fixed features of the structure (e.g., beam length) were used for comparison. However, picking the same points from different datasets for measurement is hard to achieve reliably given the discrete nature of the data capture and is arguably fraught with hard-to-quantify errors. To overcome these problems, Byrne et al. (2017) [42] proposed a point-to-point distance evaluation based on an average point-to-point distance. However, the problem remains that the geometry is not itself being checked in the absence of measured drawings, which are rarely available. Moreover, because this point-to-point distance calculation is based on the closest neighbour searching, non-uniform data distribution will cause errors to the result as well.

**Figure 5:**
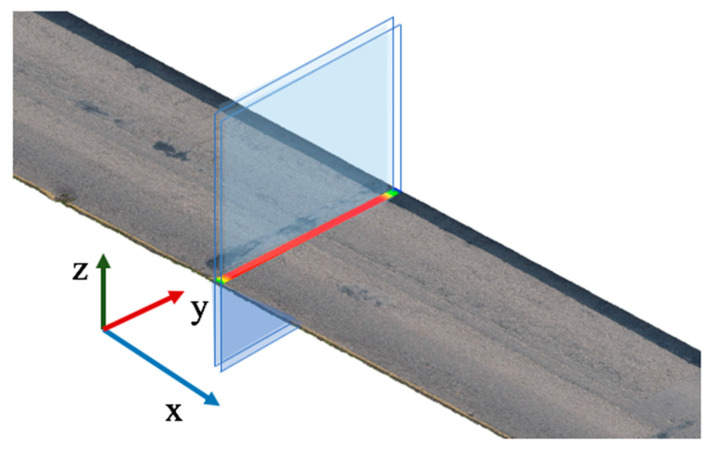
Cross-section extraction.

**Figure 6:**
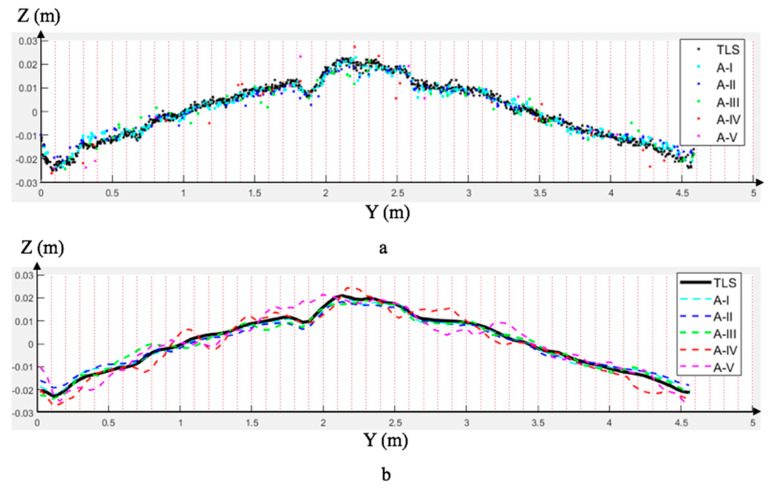
Cross-section comparison: (**a**) projected points; (**b**) fitted curves.

To resolve the problems mentioned above, a cross-section evaluation method for the accuracy assessment is proposed herein. First of all, each SfM dataset and the TLS ground truth point cloud were aligned using the ICP algorithm (Besl and McKay, 1992) [59]. Then, a cross-section (with a thickness of 5 cm in the x-direction) of the bridge deck from each dataset was manually extracted, as shown in Figure 5. After that, those points were projected to the Y–Z plane and separated into multiple intervals in the y-direction (Figure 6a). In each interval, the average Z value was calculated. By linking those points, the local surface was assembled (Figure 6b). Lastly, by measuring the difference between each SfM dataset and the TLS dataset, the Pearson correlation coefficient could be calculated through Equation (5). In that equation, $cov\left(A,B\right)$ is the covariance between two sets of mean values along the cross-section from different datasets. The terms σA and σB are the standard deviations of each set of mean values.

(5) $$\rho (A,B)=\frac{cov(A,B)}{\sigma A\sigma B}$$

### 3.4. Data Density Yield 

In some studies, the total points appearing in a reconstructed point cloud are used as a proxy to compare the quality of different reconstruction methods. For infrastructure documentation applications, such a broad approach may not encapsulate the true quality of the output, as points appearing in the background or in non-essential areas may contribute little. To determine the extent that captured data appear in the relevant portion of the point cloud, a density conversion rate (DCR) metric is proposed as a direct indicator of the yield. As shown in Equation (6), the ${AD}_{AOI}$ is the average volume density of the area of interest (AOI), in this case the bridge deck. In this equation, PN is the total number of points included in the dataset. The DCR indicates the relative value of the overall point cloud with respect to an area of interest (e.g., the bridge deck). Lower DCR values indicate a lower yield percentage with respect to all data collected.

(6) $$DCR=\frac{{AD}_{AOI}}{PN}$$

## 4. Field Study

To demonstrate the applicability and usefulness of the aforementioned metrics, a field study was undertaken. Such an approach provides insight for understanding the interaction of flight path parameter selection for bridge documentation. In this case only the bridge deck was considered as the target object.

### 4.1. Scope

The field study considered three common UAV flight path parameters: altitude, oblique angle, and overlapping rate. The Blessington bridge at Co. Wicklow, Ireland, a concrete bridge, was selected as the case study. This bridge was selected because it is outside the Dublin airport flight control area with clear surroundings and light vehicular traffic, which facilitated both UAV flights and the TLS data collection. More information about the site is presented in Section 4.3.

This section may be divided by subheadings. It should provide a concise and precise description of the experimental results, their interpretation, as well as the experimental conclusions that can be drawn.

### 4.2. Methodology

**Figure 7:**
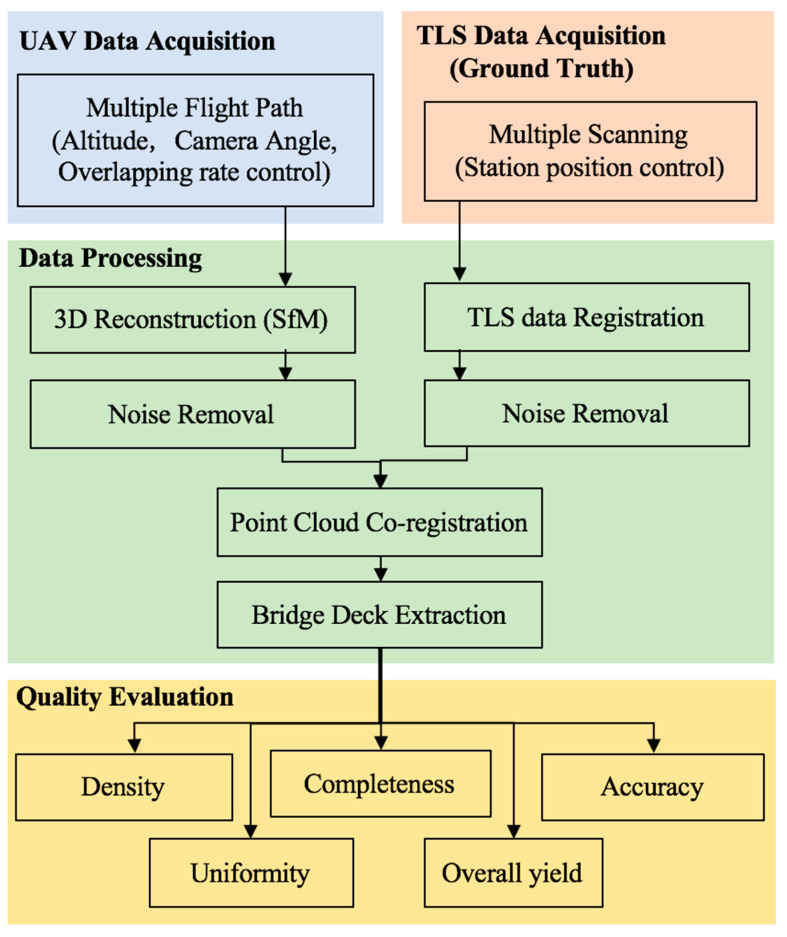
Flowchart of the data processing procedure.

The overall methodology is shown Figure 7, in which the workflow for obtaining and processing the experimental data from the UAV is shown in parallel to the acquisition and processing of the ground truth data.

**Figure 8:**
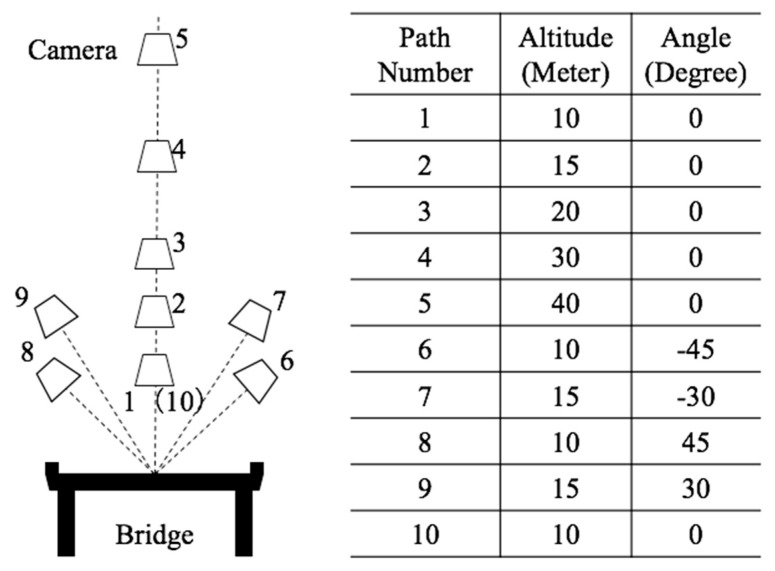
Flight path design.

The procedure includes data acquisition, processing, and evaluation of the reconstructed point cloud (Figure 7). In regard to the UAV data acquisition, multiple flight paths were designed to help determine the influence of specific parameters on the final 3D model reconstruction. As shown in Figure 8, flight paths 1–5 were situated directly over the bridge deck. These were flown at vertical offsets of 10 m, 15 m, 20 m, 30 m, and 40 m. In each of these configurations, the camera was positioned directly above the bridge deck, and the oblique angle (the angle between the camera centre line to the bridge deck’s normal direction) was 0°. These flights considered the impact of elevation.

To determine the effect of the oblique angle, two flight paths were undertaken (flight path 9, Figure 8). These were conducted along each side of the bridge, with one oriented at 45° and one at 30° from the bridge deck. In both cases, the offset distance was approximately 15 m from the deck centre and thus captured different vantage points. For flight paths 1–9, the image overlapping rate was above 80%. In addition to those images, path 10 was flown along the previous path, path 1, with an overlapping rate higher than 90%. Table 2, Table 3, Table 4 and Table 5 demonstrate how each of these flight paths were used singly and in combination to create 15 distinct datasets. Dataset Groups A and B used images from single flight paths 1–9 to analyse the effect of altitude and angles. Dataset Group C used multiple flight paths to evaluate the effect of different combination strategies. Dataset Group D used images from flight path 10. Dataset D-I used all images as the input, while D-II and D-III were generated using only the second (D-II) or third (D-III) image in the acquisition sequence. Thus, three different datasets in Group D were created to check the effect of the overlapping rate with respect to image acquisition speed.

After the image acquisition, a standard SfM 3D reconstruction process and noise reduction process was applied using methods previously introduced by the authors (Chen et al., 2017; Chen et al., 2018 [60]). Then, to ensure that the same section of the bridge was compared, each 3D point cloud was aligned with its accompanying TLS dataset through the ICP algorithm (Besl and McKay, 1992). After data alignment, the bridge deck was extracted in each dataset to evaluate the quality and accuracy of each model on a local (per-point) basis. For this, five metrics were employed in the form of point density (Equation (1)), point uniformity (Equation (3)), completeness of the reconstruction (Equation (4)), geometric accuracy (Equation (5)), and data yield (Equation (6)).

### 4.3. Experimental Set Up

**Figure 9:**
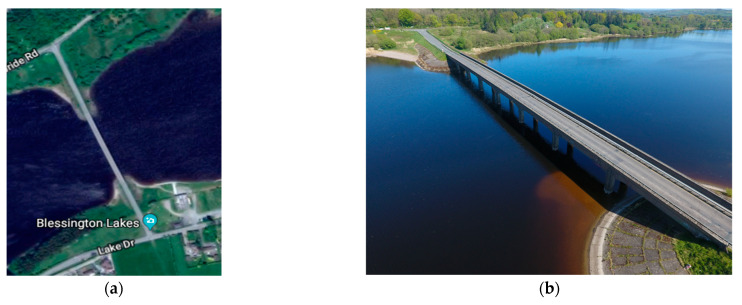
Blessington bridge and surrounding environs: (**a**) satellite image; (**b**) aerial image.

To investigate flight path optimization, an experiment was conducted using the Blessington bridge in County Wicklow, Ireland. The bridge is constructed of reinforced concrete, is approximately 130 m long and 8 m wide, and is typically situated 10 m above the water level (Figure 9).

**Figure 10:**
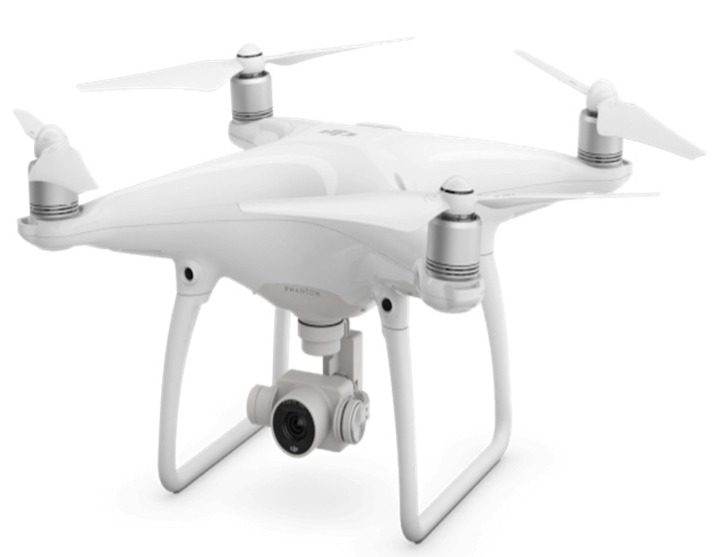
UAV with 4k camera.

**Figure 11:**
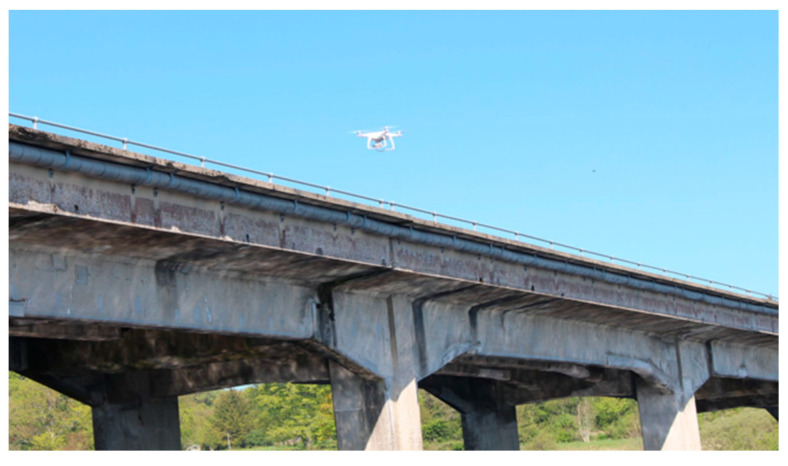
Image acquisition (UAV is shown above front right support).

A DJI Engineering quadrotor Phantom 4 was used for the experiment. The UAV was equipped with a 4K camera (3000 × 4000 pix) and a 3-axis gimbal, as shown in Figure 10 and Figure 11. The total cost for the system was about EUR 1500. The take-off, image capture, and landing operations were manually controlled by a remote pilot through a first-person-view camera with a mandated second operator to help ensure obstacle avoidance.

### 4.4. Data Processing

The 3D reconstruction process was performed in the commercial software PhotoScan (Agisoft, 2017) with GPS tagging. In the software, both the image alignment accuracy and the dense point reconstruction quality were set to high. The reconstructions were processed on a Dell XPS 15 laptop (i7 GPU, 16 Gb RAM); the results are reported in Section 6. Point cloud registration, manual bridge deck extraction, and density calculations were achieved through the open-source software CloudCompare2.11.3 (CloudCompare, 2017) [61], and Equations (3)–(7) were implemented in MatLab.

### 4.5. TLS Data Collection

**Figure 12:**
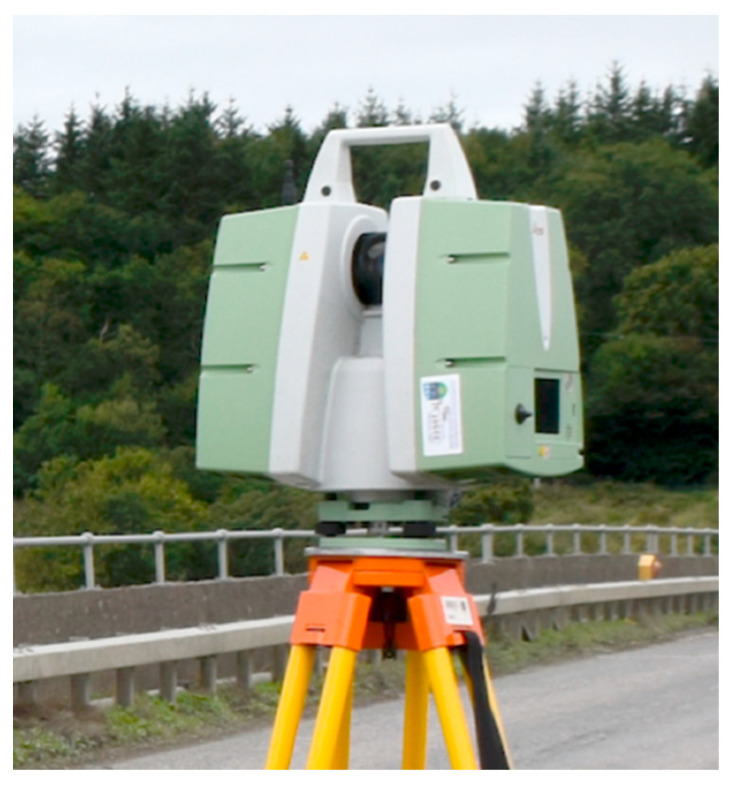
P20 laser scanner.

**Figure 13:**
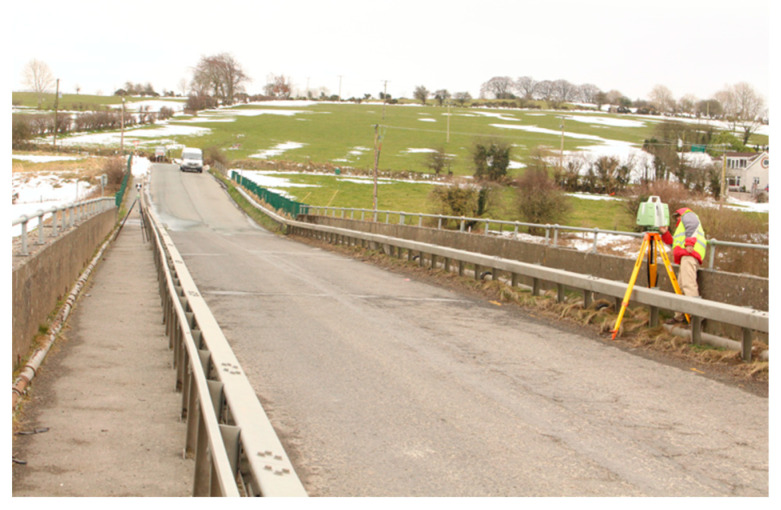
TLS data acquisition.

**Figure 14:**
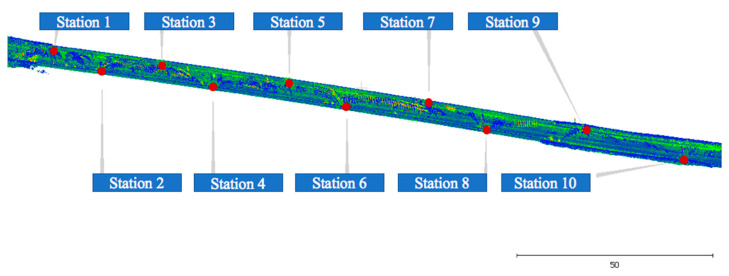
Distribution of 10 TLS stations.

The TLS data to be used for benchmarking were collected with a Leica Scan Station P20 terrestrial laser scanner (Figure 12 and Figure 13). The bridge desk was captured from a total of 10 scan stations (see Figure 14) along the side path of the bridge. The resolution was set as 6.1 mm at 10 m, resulting in a sampling step of 5 mm. That data collection took approximately 3 h by one surveyor including logistics and scanner set up. The scanning only required about 7 min per scan station including data and target capture. Scan co-registration was performed by using Leica’s proprietary software Cyclone (V9.1). The final dataset contained approximately 270 million points. The local geometric accuracy was measured using TLS as the ground truth. TLS data have high resolution and accuracy at close distances via a single scan; multiple long-distance surveys as would be required for global accuracy would have cumulative global errors introduced by the registration process.

## 5. Results

### 5.1. Collected Data

The image acquisition process was conducted in the early morning to minimize vehicular-based occlusions. During the UAV imagery acquisition process, 526 images were captured across the 10 flight paths. Of the 55 min required for imagery data collection, 14 was for site checks, take-offs, reversals, and landings (see Table 6 for more details). The highest ground resolution (GR) achieved was 3.71 mm/pixel. The individual flights ranged from 2 to 10 min yielding as few as 21 and as many as 143 images at data capture rates of 8.7 to 15.7 images per minute but at a constant overlapping rate. More details are shown in Table 6.

### 5.2. Error Sources

A key aim of this paper is to provide a better understanding of how different UAV flight paths impact the quality of imagery-based point clouds for the inspection of bridge decks and similar infrastructure. To that end, flight paths were designed with pre-specified altitudes and offset distances from the bridge. However, the equipment’s on-board GPS system has an advertised hover accuracy of ±0.5 m in the vertical direction and ±1.5 m in the horizontal direction (DJI, 2019). Furthermore, the field conditions included wind effects. By checking the camera pose estimation results, a presence drift was verified. For example, flight path 1 and flight path 10 were intended to have identical altitudes of 10 m. In reality, the average capture distance for path 1 was 9.5 m, while that for path 10 was 10.5 m. While such differences affected the ground resolution of the captured images, the general trends being reported herein were not impacted.

**Figure 15:**
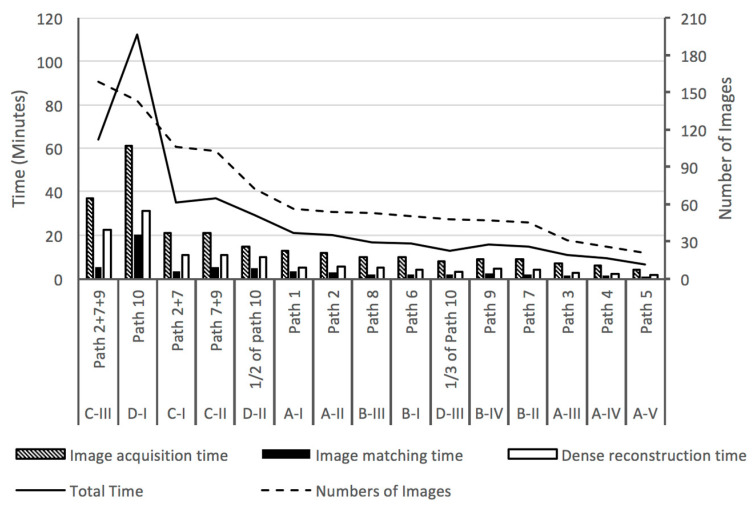
Image number and post-processing time.

Characteristics of the SfM point clouds derived from those images are shown in Table 7. Generally, the total processing time related to the quantity of input images (Figure 15). However, dataset C-III used less processing time than the less-populated dataset D-I. A possible reason is that the multiple flight paths were parallel to each other. Thus, overlapping between images occurred both in the horizontal and vertical directions, which appears to have decreased the feature matching time (Byrne et al., 2017) in a different experimental arrangement.

### 5.3. Density and Uniformity Comparison

**Figure 16:**
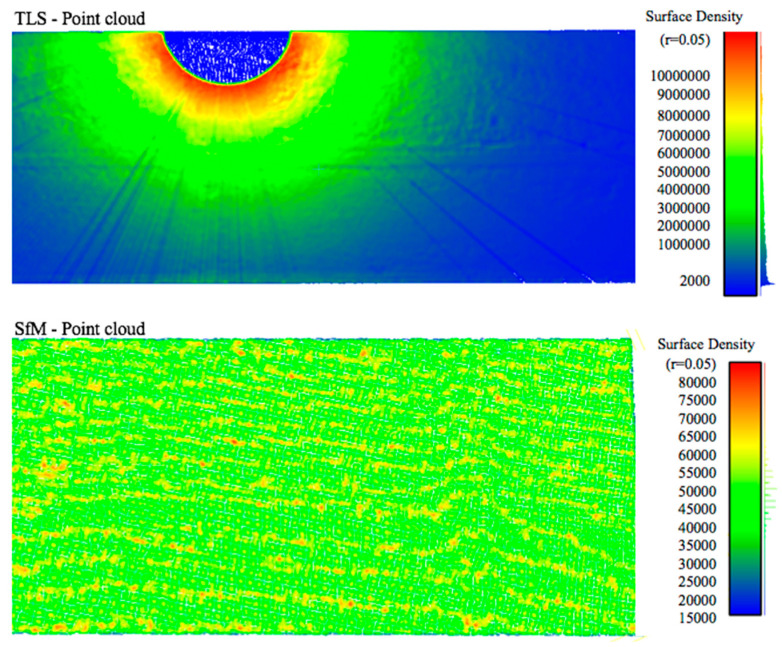
Data density maps of TLS and SfM point cloud.

As expected, the TLS dataset had a point density with a radial distribution, with the scanner at its centre producing higher-density point areas closer to the scanner. Lower-density strips are an artefact of cars or pedestrians passing in front of the scanner. Within allowable time constraints, these were minimized by re-performing the scans. In contrast, the SfM point cloud exhibits a largely uniform point distribution across the study area interspersed with waves of slightly lower density strips, as shown in Figure 16. This comparative homogeneity of the data offers a constant data resolution across the entire structure and reduces post-processing difficulties, as previously mentioned. To further understand how the flight path setting interacted with general point density and uniformity, the volume-based density calculation method introduced in Section 3.1 was applied to all 15 datasets (Table 7). The results are shown in Table 8.

As expected, the results shown in Table 8 demonstrate a significant correlation between the flight altitude and data density, with lower flights generating more uniform datasets (approximately 1% improvement per meter in RSD). The linear overlapping rate also affected the density. In the D Test series, as the overlap rate increased from 66% to 90%, the data density increased by about 10%, while in the A Test series the density increased by more than 227% when the altitude decreased from 40 m to 10 m.

As shown in Table 9, when comparing B Test series outputs to A Test series outputs, datasets obtained with narrower oblique angles at the same altitude led to denser point clouds than those collected with wider one. Also shown in Table 9, datasets with similar ground resolutions (B-I, B-III, and A-II) exhibited similar average densities.

Importantly, the C Test series showed that, instead of increasing the final point density, adding more flight paths from various angles decreased the final point cloud density. Based on work by Byrne et al., the extra images may be providing rich geometric information, which would then allow for the better detection of invalid points or noise and their subsequent removal as part of the reconstruction process. This concept of quality over quantity is further explored in Section 6.1.

**Figure 17:**
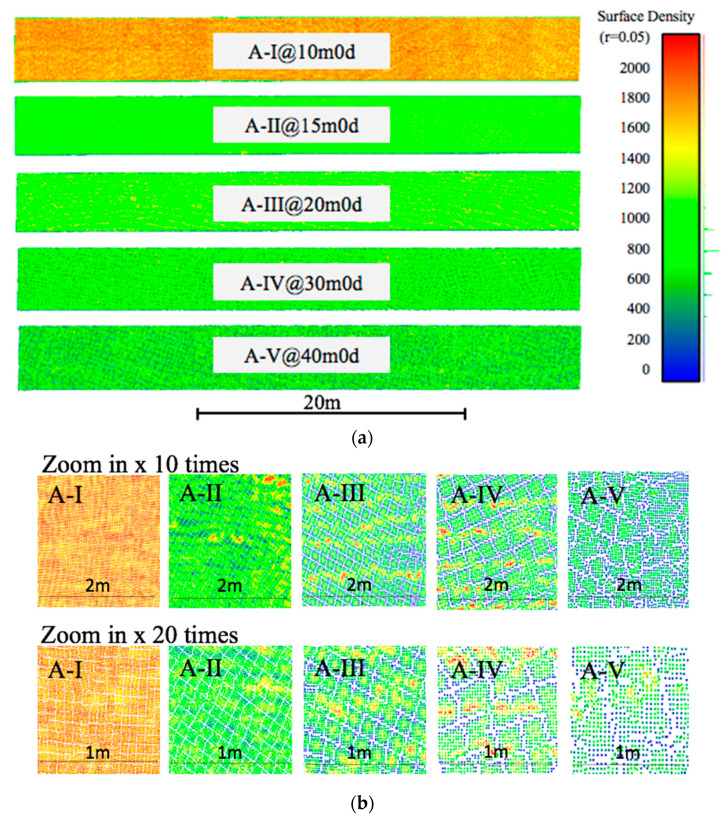
Density maps for the test series: (**a**) bridge deck data coverage; (**b**) bridge deck data coverage.

To better understand the RSD changes in the SfM point cloud, a density map was generated (Figure 17) for the A Test series. At each fight altitude, different data density patterns appear. Close ups (10 and 20 times) illustrate that those patterns segmented the point cloud into numerous irregular grids, and in the boundary of each grid, points are missing (Figure 17). The grid size and the gap width are highly related to flight altitude (Figure 17). A probable reason is that with the increasing altitude, the ground resolution of each pixel increases correspondingly. As the pixel is the smallest unit for feature detection, ground resolution will be directly affected in the feature matching process (Verhoeven et al., 2015; Apollonio et al., 2014) [62,63]. After feature matching, the dense reconstruction process occurs through MVS algorithms to generate denser patches around matched seed features (Shao et al., 2016 [64]). In the detailed inspection of the data, around each patch is a gap where no data exist and which reduces the overall average density. The size of the gaps increases with altitude (Figure 17).

### 5.4. Completeness Comparison

The aforementioned gaps are treated as incomplete areas and quantified by Equation (4). The average point-to-point distance β_ave_ of each dataset was selected as the threshold for mesh generation. Areas where this threshold was exceeded were considered as incomplete. Table 10 demonstrates that for the particular equipment and the specific bridge in this field study, the single path datasets ranged in completeness from just over 66% to nearly 77%—generally producing better ones at lower altitudes. The fixed angle and different altitudes in Group A showed a U-shaped distribution in completeness. With increasing altitude, the completeness level dropped quickly in the beginning. Then, above 20 m it increased again but more slowly. The highest completeness was achieved by the lowest flight path centred over the pavement’s centre. When the 80% overlapping rate in A-I was increased to 90% at the same altitude in D-I, the completeness rate nudged slightly higher but certainly nothing close to proportional for the additional quantity of data being collected and processed.

Dataset Group B showed that depending upon the oblique angle much greater completeness can be achieved with significantly less data. In this case the completeness was nearly 10% more even in the absence of nearly a third of the data. Interestingly, when flight patterns were mixed other complexities arose, as shown in Group C where the completeness levels were less than other groups, as measured herein. The multiple flight paths caused a mixing of the grid layouts, thereby resulting in a range of gap sizes (Figure 17). When processed according to the procedure described in Section 3.2, a small threshold was selected, which was then used to calculate the completeness rate. Consequently, the non-uniform gaps introduce an artefact into the dataset that influences the calculation. Therefore, this must be considered as a limitation of this newly proposed metric.

### 5.5. Geometry Accuracy Comparison

A geometric accuracy assessment was conducted by comparing a cross-section of each SfM point cloud to the equivalent portion of the TLS point cloud. In this test, the cross-section was divided into 200 intervals. The mean altitude of each interval was calculated and compared through the method introduced in Section 4.2.

**Figure 18:**
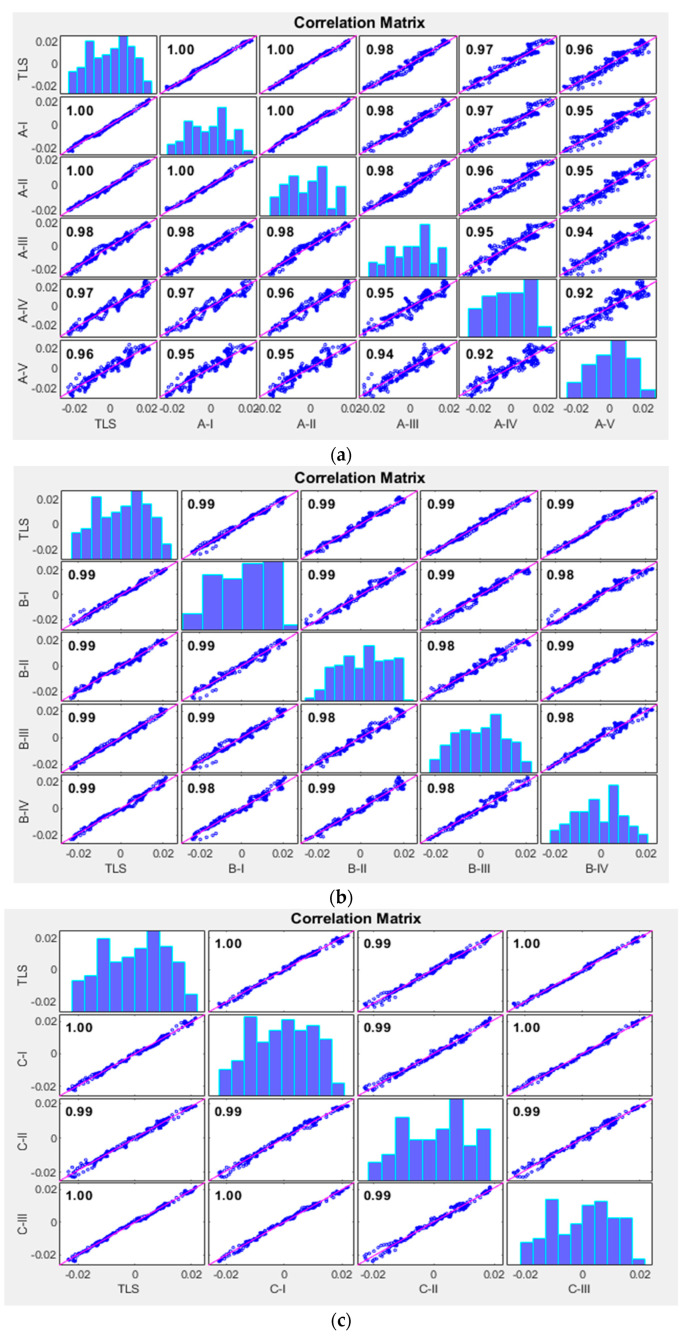

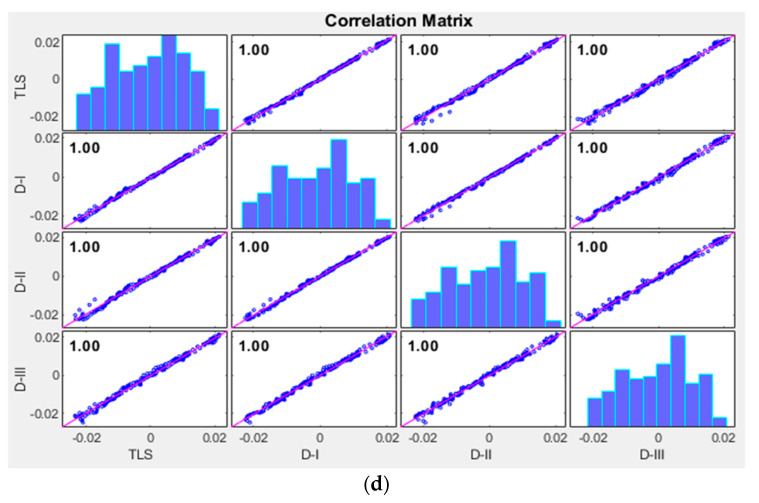
Correlation matrix of accuracy assessment: (**a**) correlation matrix of Group A; (**b**) correlation matrix of Group B; (**c**) correlation matrix of Group C; (**d**) correlation matrix of Group D.

As visible in Figure 18 and Table 11, the geometric accuracy was affected by all parameters. As expected, the Group A test series showed a linear improvement with lower altitudes. Similarly, the overlapping rate and oblique angle had direct effects on the accuracy. Using multiple view angles increased the geometric accuracy compared to processing each angle separately. In summary, as expected, the best overall results were achieved at lower altitudes, smaller angles, and higher overlapping rates.

### 5.6. Data Yield

The DCR results are shown in Table 12. According to the Group A tests, lower altitudes have higher DCRs, as would be expected peripheral information such as the river or its banks is not being captured. Test Series B shows that the oblique angle will also decrease the DCR, this because the oblique angle captures more of the bridge’s side view. In Test Series C, even though the total number of points (PN) were similar across the data series, the average point density (AD) and the final data yield (DCR) differed significantly. Capturing the bridge’s side data negatively impacted these two metrics. Test Series D shows that, the higher overlapping rate improved the PN significantly. However, the AD did not change much and the DCR decreased when the overlapping rate increased, which means the higher overlapping rate decreased the efficiency of point utilization.

## 6. Discussion

### 6.1. Flight Path Optimization

**Figure 19:**
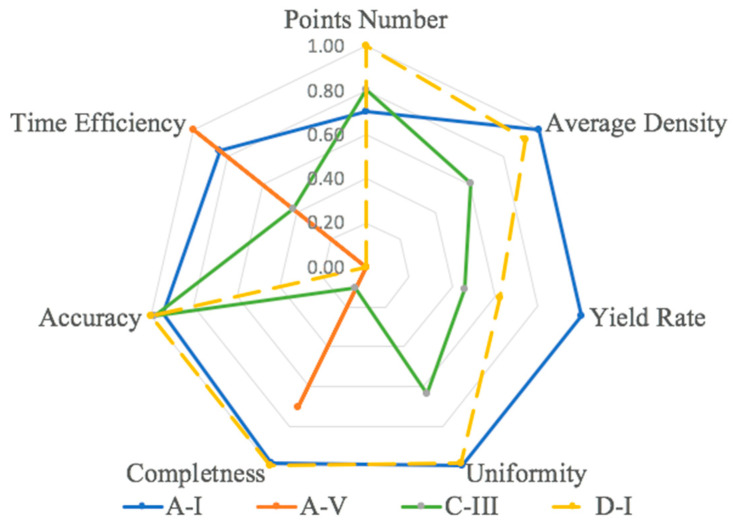
Radar map for performance comparison.

To provide guidance for flight path planning, each category of data analysed in Table 7, Table 8, Table 9, Table 10, Table 11 and Table 12 was normalized by the highest value achieved across the 15 datasets and compiled in Table 13. Those datasets with the best performance in at least one metric were further analysed in a seven-pronged radar map to show a more holistic performance across the various metrics (Figure 19). Unlike previous research using the total point numbers or the average density as a unique standard to evaluate the reconstruction performance, herein seven different metrics are proposed and compared. Both from Table 13 and Figure 19, highly distinctive patterns can be observed.

As such, proper flight path selection must be informed by the survey’s purpose. For example, Dataset A-I, which had the closest survey distance (9.5 m) and no offset oblique angle, produced the highest average density, yield rate, and uniformity, demonstrating that this flight path can generate a well-distributed point cloud. Additionally, it also had a good balance in completeness, geometric accuracy, and time efficiency. Dataset D-I illustrated that by adding more images to increase the overlapping the completeness and accuracy level could be increased. However, this improvement is costly. To improve the completeness by 1%, when compared to D-II, D-I tripled the time cost in image acquisition and post-processing. In some surveys, rapid assessment through shorter flight times and limited processing periods is important, such as after nature disasters. In those cases, if the bridge deck area is the focus of concern, then flying at a higher altitude directly over the bridge (e.g., path A group) may be the most appropriate choice at the cost of accuracy and density.

The evaluation concepts of accuracy and completeness, as well as point yield, introduced in this paper, provide a more holistic and, arguably, more rigorous approach to UAV-based imagery acquisition for bridge documentation. In fact, the experimental work demonstrates that maximizing point density may actually be counterproductive to obtaining cost-effective and comprehensive point clouds depending upon the position of the UAV with respect to the areas targeted for documentation.

### 6.2. UAV Photogrammetry vs. TLS

TLS is often proposed as an alternative solution for bridge documentation. For the quality evaluation purpose, this section compares the best SfM point clouds achieved in the experiments that were achieved by TLS. As mentioned in some other studies (Hallermann et al., 2015; Chen et al., 2018 [65]), the advantages of UAV imagery data collection include high efficiency and low costs. In this experiment, even with multiple flight paths (10 paths), the entire flight time was less than one hour—only a third of the TLS data collection time. In this instance, the post-processing times were almost the same for the UAV images’ SfM reconstruction and the TLS data’s co-registration (Table 14). However, as illustrated in Figure 19, the SfM post-processing time is highly dependent upon image quantity. If the datasets have large amounts of sky and water, image matching becomes harder and more time consuming. However, if the imagery is collected via video, a limited number of frames can be automatically selected to restrict the image matching process during reconstruction, as explained by Byrne et al. (2017). In contrast, the TLS data co-registration time is largely linear, more predictable, and can be minimized based on reducing the number of scan station locations. Additionally, the UAV-SfM system used in this study cost only 10% of the cost of the TLS system budget and generated a competitive result (Table 14). However, this figure does not include UAV training, permitting, or insurance costs.

This paper’s experimental results for documenting a bridge deck demonstrated that a well-designed flight path can achieve two-thirds of the average density of the TLS result, with a geometric difference as little as 3 mm. While this figure is important, what is arguably of greater concern for further post-processing is the uniformity of the point cloud. The UAV-SfM point cloud is much more uniform than the TLS result (RSD of 5.56–25.6% vs. 73.12%) and with almost no low-density pockets. Moreover, with the designed metric and strict threshold, the completeness level of TLS is only 7.49%. That means only 7.49% of the entire survey area was covered by well-distributed, high-density points. In contrast, the UAV-SfM method easily achieved more than 50%. The higher completeness and better uniformity of the SfM point cloud has many benefits for inspections, such as (1) less unknowns and (2) more ability to obtain consistent post-processed objects, as the input is more uniform. However, the UAV method is highly vulnerable to the weather. Wind especially affects flight path quality by causing the camera to shake and the UAV to drift—both impacting the final quality. Sunlight was also shown to have some impacts. Thus, when designing a proper flight path for a specified quality, those issues should be considered ahead of time.

## 7. Conclusions

To optimize UAV-SfM bridge deck inspection or similar applications, flight path design and data capture considerations in terms of altitude, angle of capture, overlapping rate, and combined flight paths were explored. To evaluate the various outcomes, this paper proposed a suite of seven evaluation metrics to check the variance of point cloud quality and overall efficiency in the form of the total number or points, average density, uniformity, yield rate, completeness, geometry accuracy, and time efficiency. In the presented case study of the Blessington Bridge, bridge deck geometry was acquired from 10 different flight paths from which 15 groups of point cloud datasets were assembled as generated through an SfM method. Evaluation of these 15point clouds established that both altitude and oblique angle significantly affected the point density and uniformity.

Several major conclusions can be drawn from this study. First, irrespective of the individual and combined parameters, the SfM process resulted in point groupings in semi-irregular grids with clearly identifiable gaps between point groups. The size of both the grids and the gaps increased at higher flight altitudes. Multiple flight paths resulted in a combination of the individual grid patterns from the specific flight paths, which decreased the general completeness rate but improved the overall geometric accuracy. The best completeness (77%) was achieved by a single flight path with the lowest altitude (9.5 m) and an 80% overlapping rate. Next, while the overlapping rate strongly affects the total number of points, it only weakly impacts the average density of the portion of the point cloud representing the deck surface, thereby negatively impacting the time efficiency without strongly improving the data yield rate. However, in this study a minimum overlapping rate of 66% was found to be needed to successfully achieve the SfM reconstruction process.

Additionally, this research suggests that there is not a unique solution for UAV bridge deck surveys due to the complex relationship between the flight path settings and the specific survey objectives’ (e.g., accuracy, completeness, and economy) strong influence on the optimal data capture strategy (Figure 19). For example, if high accuracy is the goal, using a lower altitude, smaller angle, and higher overlapping rate can achieve better results than other flight path combinations. Finally, in the case study presented herein, the UAV-SfM method demonstrated some critical advantages over TLS documentation, including time efficiency, general cost, and data uniformity, but at the expense of point density and some accuracy.

## Figures and Tables

**Table 1:** Sampling of UAV inspection-related research.

UAV Type	Sensor	Data Output	Purpose	Researcher
Multirotor	Laser scanner	2D point cloud	Forest survey: tree diameter measurements	(Chisholm et al., 2013) [18]
Multirotor	Laser scanner	3D point cloud	Building survey: house dimension measurement	(Roca et al., 2015) [20]
Multirotor	Laser scanner and camera	3D point cloud	Forest survey: quality comparison between laser and SfM for copy measurement	(Wallace et al., 2016) [21]
Multirotor	Laser scanner	3D point cloud	Point-cloud-based deep learning and UAV path planning for surface defect detection of concrete bridges	(Bolourian et al., 2022) [22]
Multirotor	Camera	2D images	Bridge survey: 3D model generation for bridge	(Hallermann and Morgenthal, 2014) [23]
Multirotor	Camera	2D images	Bridge inspection: crack detection from distortion images	(Ellenberg et al., 2016) [24]
Multirotor	Camera	2D images	Building inspection: crack detection from hybrid image	(Kim et al., 2017) [25]
Multirotor	Camera	2D images	Bridge inspection: using Digital Image Correlation (DIC) technique for crack measurement	(Reagan and Sabato, 2017) [26]
Multirotor	Camera (thermal)	2D images	Bridge inspection: delamination mapping for concrete bridge deck	(Escobar-Wolf et al., 2017) [27]
Multirotor	Camera (thermal)	2D images	Bridge inspection: delamination measurement for concrete bridge deck	(Omar and Nehdi, 2017) [28]
Multirotor	Camera (thermal)	2D images	STUDY FOR UAS-ASSISTED BRIDGE INSPECTIONS	(E.T. Bartczak, 2023) [29]
Multirotor	Camera	3D point cloud	Building survey: 3D model generation for aging structures	(Hallermann, et al., 2015) [30]
Multirotor	Camera	3D point cloud	Bridge survey: 3D model generation for historical bridge	(Hallermann et al., 2015) [31]
Multirotor	Camera	3D point cloud	Bridge survey: 3D model generation for timber truss bridge	(Khaloo et al., 2017) [32]
Multirotor	Camera	3D point cloud	Building survey: 3D model generation from image in sequence	(Qu et al., 2018) [6]
Multirotor	Camera	3D point cloud	Bridge survey: 3D model accuracy evaluation	(Calì and Ambu, 2018) [33]
Multirotor and fixed-wing	Camera	3D point cloud	Road inspection: accuracy comparison of TLS and SfM	(Ruggles et al., 2016) [20]
Multirotor	Camera	3D point cloud	Optimal UAV image overlap for photogrammetric 3D reconstruction of bridges	(F wang et al., 2022) [34]
Multirotor	Camera	3D point cloud	Geometric shape measurement and its application in bridge construction based on UAV and terrestrial laser scanner	(Yitian Han et al., 2023) [35]

**Table 2:** Altitude comparison.

Dataset Name	Input Data Source	Designed Altitude (m)
A-I	Path 1	10
A-II	Path 2	15
A-III	Path 3	20
A-IV	Path 4	30
A-V	Path 5	40

All flights directly above the bridge deck’s centre line.

**Table 3:** Oblique angle comparison.

Dataset Name	Input Data Source	Designed Angle (Degree)	Designed Altitude (m)	Designed Offset (m)	Distance to Centre Line (m)
B-I	Path 6	−45	10	10	14.14
B-II	Path 7	−30	15	10	20
B-III	Path 8	45	10	10	14.14
B-IV	Path 9	30	15	10	20

**Table 4:** Path combination comparison.

Dataset Name	Input Data Source	Path Combination
C-I	Path 2 + 7	Top + one side
C-II	Path 7 + 9	Two sides
C-III	Path 2 + 7 + 9	Top + two sides

**Table 5:** Overlapping rate comparison.

Dataset Name	Input Data Source	Overlapping Rate
D-I	Path 10	90%
D-II	50% of Path 10 data	83%
D-III	33% of Path 10 data	66%
D-IV	25% of Path 10 data	50%

**Table 6:** UAV image acquisition result.

Flight Path	Designed Altitude (Meter)	Designed Oblique Angle (Degree)	Images Acquired	Ground Resolution (mm/pixel)	Flying Time (Minutes)	Data Capture Rate (Images/ Minute)
1	10	0	56	3.71	5	11.2
2	15	0	54	5.88	4	13.5
3	20	0	31	8.71	3	10.3
4	30	0	26	13.4	3	8.7
5	40	0	21	18.5	2	10.5
6	10	−45	50	6.49	4	12.5
7	15	−30	45	7.26	3	15.0
8	10	45	53	5.73	4	13.3
9	15	30	47	7.44	3	15.7
10	10	0	143	4.09	10	14.3

**Table 7:** Three-dimensional point cloud reconstruction results.

Dataset Name	Input Data Source	Number of Images	Image Acquisition Time	Image Matching Time	Total Points Number	Reconstruction Time	Total Time
A-I	Path 1	56	5	2.9	42,849,707	5.3	13.3
A-II	Path 2	54	4	2.8	29,810,855	5.4	12.3
A-III	Path 3	31	3	1.4	16,800,275	2.6	7.0
A-IV	Path 4	26	3	1.2	12,725,012	2.1	6.3
A-V	Path 5	21	2	0.8	9,550,978	1.9	4.6
B-I	Path 6	50	4	1.8	22,761,870	4.2	10.0
B-II	Path 7	45	3	1.6	23,503,173	4.3	9.0
B-III	Path 8	53	4	1.7	28,807,016	4.9	10.5
B-IV	Path 9	47	3	2.0	24,261,326	4.5	9.5
C-I	Path 2 + 7	106	7	3.4	46,732,039	10.9	21.3
C-II	Path 7 + 9	103	6	5.0	41,138,599	11.0	21.9
C-III	Path 2 + 7 + 9	159	10	4.9	47,549,256	22.4	37.3
D-I	Path 10	143	10	20.3	56,929,560	31.1	61.3
D-II *	1/2 of path 10 images	72	5	4.7	47,219,880	9.8	19.5
D-III	1/3 of path 10 images	48	3.3	2.0	38,213,629	3.0	8.2
D-IV	1/4 of path 10 images	36	2.5	Failed	Failed	Failed	Failed

* Every other image was used to achieve the reduced dataset.

**Table 8:** Data density and uniformity comparison.

Test A	Designed Altitude (m)		AD (Points/m^3^)	RSD
A-I	10		298,474	5.56%
A-II	15		118,192	9.34%
A-III	20		54,765	14.17%
A-IV	30		21,905	20.38%
A-V	40		13,117	25.61%
Test B	Designed Angle (degree)	Designed Altitude (m)	AD (points/m^3^)	RSD
B-I	−45	10	111,861	8.40%
B-II	−30	15	77,574	10.27%
B-III	45	10	127,153	7.58%
B-IV	30	15	75,954	8.50%
Test C	Combination		AD (points/m^3^)	RSD
C-I	Top + one side		219,979	16.35%
C-II	Two sides (±30°)		75,205	13.22%
C-III	Top + two sides		187,203	12.87%
Test D	Overlapping Rate		AD (points/m^3^)	RSD
D-I	90%		277,159	5.80%
D-II	83%		263,239	6.02%
D-III	66%		251,963	5.96%
Benchmark	Stations		AD (points/m^3^)	RSD
TLS	10		4,163,133	73.12%
Best Results	Path Setting		AD (points/m^3^)	RSD
A-I	10 m		298,474	5.56%
B-III	45 degrees		127,153	7.58%
C-I	Top + one side		219,979	16.35%
D-I	90% overlap		277,159	5.80%

**Table 9:** Comparison of oblique angle impact.

Datasets	Altitude	Angle	GR	AD	RSD
A-I	10	0	3.71	298,474	5.56%
B-I	10	−45	6.49	101,861	8.40%
B-III	10	45	5.73	127,153	7.58%
A-II	15	0	5.88	118,192	9.34%
B-II	15	−30	7.26	77,574	10.27%
B-IV	15	30	7.44	75,954	8.50%

**Table 10:** Comparison of completeness.

Altitude Change Effect Datasets	Altitude	Density	β_ave_ (mm)	Completeness
A-I *	10	298,474	5.4	76.80%
A-II	15	118,192	8.5	69.30%
A-III	20	54,765	12.8	66.41%
A-IV	30	21,905	19.4	68.25%
A-V	40	13,117	26.8	68.75%
Angle Change Datasets	Altitude	Density	β_ave_ (mm)	Completeness
B-I	−45	111,861	8.3	56.10%
B-II	−30	77,574	10.3	64.72%
B-III	45	127,153	7.5	59.85%
B-IV	30	75,954	10.2	62.49%
Flight Path Arrangement Effect Datasets	Path location	Density	β_ave_ (mm)	Completeness
C-I	Top + one side	219,979	5.7	48.52%
C-II	Two sides	75,205	10.5	59.54%
C-III	Top + two sides	187,203	6.1	51.59%
Overlapping Rate Effect Datasets	Overlapping	Density	β_ave_ (mm)	Completeness
D-I	90%	277,159	5.2	77.26%
D-II	83%	263,239	5.6	76.84%
D-III	66%	251,963	5.9	76.09%
Benchmark Datasets	Stations	Density	β_ave_ (mm)	Completeness
TLS	10	4,163,133	1.2	7.49%

* 80% overlapping rate.

**Table 11:** Comparison of geometric accuracy based on flight parameters.

Altitude Effect Datasets	Altitude (m)	Correlation Coefficient
Entry 1	data	data
A-I	10	0.9962
A-II	15	0.9952
A-III	20	0.9849
A-IV	30	0.9711
A-V	40	0.955
Altitude Effect Datasets	Angle (degree)	Correlation Coefficient
B-I	−45	0.9935
B-II	−30	0.9903
B-III	45	0.9936
B-IV	30	0.9919
Flight Path Effect Datasets	Path location	Correlation Coefficient
C-I	Top + one side	0.9962
C-II	Two sides	0.9945
C-III	Top + two sides	0.9978
Overlapping Rate Effect Datasets	Overlapping	Correlation Coefficient
D-I	90%	0.9986
D-II	83%	0.9969
D-III	66%	0.9945

**Table 12:** Data yield rate.

**Test A**	**Altitude (m)**	**PN**	**AD**	**DCR**
A-I	10	42,849,707	298,474	6.97 × 10^−3^
A-II	15	29,810,855	118,192	3.96 × 10^−3^
A-III	20	16,800,275	54,765	3.26 × 10^−3^
A-IV	30	12,725,012	21,905	1.72 × 10^−3^
A-V	40	9,550,978	13,117	1.37 × 10^−3^
A-I	10	42,849,707	298,474	6.97 × 10^−3^
**Test B**	**Angle(degree)**	**PN**	**AD**	**DCR**
B-I	-45	22,761,870	111,861	4.91 × 10^−3^
B-II	-30	23,503,173	77,574	3.30 × 10^−3^
B-III	45	28,807,016	127,153	4.41 × 10^−3^
B-IV	30	24,261,326	75,954	3.13 × 10^−3^
**Test C**	**Combination**	**PN**	**AD**	**DCR**
C-I	Top + one side	46,732,039	219,979	4.71 × 10^−3^
C-II	Two sides (±30°)	41,138,599	75,205	1.83 × 10^−3^
C-III	Top + two sides	47,549,256	187,203	3.94 × 10^−3^
**Test D**	**Overlapping Rate**	**PN**	**AD**	**DCR**
D-I	90%	56,929,560	277,159	4.87 × 10^−3^
D-II	83%	47,219,880	263,239	5.57 × 10^−3^
D-III	66%	38,213,629	251,963	6.59 × 10^−3^
**Benchmark**	**Stations**	**PN**	**AD**	**DCR**
TLS	10	270,276,582	4,163,133	1.54 × 10^−2^

**Table 13:** Normalized results.

Dataset Name	Points Number	Average Density	Yield Rate	Unifor-mity	Completeness	Geometric Accuracy	Time Efficiency
A-I	0.70	1.00	1.00	1.00	0.98	0.94	0.85
A-II	0.43	0.37	0.46	0.81	0.72	0.92	0.87
A-III	0.15	0.15	0.34	0.57	0.62	0.69	0.96
A-IV	0.07	0.03	0.06	0.26	0.69	0.37	0.97
A-V	0.00	0.00	0.00	0.00	0.70	0.00	1.00
B-I	0.28	0.35	0.63	0.86	0.26	0.88	0.91
B-II	0.29	0.23	0.34	0.77	0.56	0.81	0.92
B-III	0.41	0.40	0.54	0.90	0.39	0.89	0.90
B-IV	0.31	0.22	0.31	0.85	0.49	0.85	0.91
C-I	0.78	0.72	0.60	0.46	0.00	0.94	0.71
C-II	0.67	0.22	0.08	0.62	0.38	0.91	0.70
C-III	0.80	0.61	0.46	0.64	0.11	0.98	0.42
D-I	1.00	0.93	0.62	0.99	1.00	1.00	0.00
D-II	0.80	0.88	0.75	0.98	0.99	0.96	0.74
D-III	0.60	0.84	0.93	0.98	0.96	0.91	0.94

**Table 14:** UAV-SfM vs. TLS.

Features	UAV-SfM	TLS
Data acquisition time	10 min for image capturing (of dataset D-I)	170 min for 10 scan locations
Point cloud generation time	52 min for image capturing (of dataset D-I)	50 min for 10 dataset registration and irrelevant point removal
Point numbers	57 million points	270 million points
Equipment cost	USD 5000 including the UAV platform and reconstruction software	USD 50,000 including the Leica P-20 and post-processing software

## Data Availability

All data and codes that support the findings of this study are available from the corresponding author upon reasonable request.
